# Detecting Spatially Localized Exciton in Self-Organized InAs/InGaAs Quantum Dot Superlattices: a Way to Improve the Photovoltaic Efficiency

**DOI:** 10.1186/s11671-017-2218-2

**Published:** 2017-07-11

**Authors:** Maher Ezzedini, Tarek Hidouri, Mohamed Helmi Hadj Alouane, Amor Sayari, Elsayed Shalaan, Nicolas Chauvin, Larbi Sfaxi, Faouzi Saidi, Ahmed Al-Ghamdi, Catherine Bru-Chevallier, Hassen Maaref

**Affiliations:** 1 Université de Monastir, Laboratoire de Micro-Optoélectronique et Nanostructures (LMON), Faculté des Sciences, 5019 Monastir, Tunisia; 2 Institut des Nanotechnologies de Lyon (INL)-UMR5270-CNRS, INSA-Lyon, 7 avenue Jean Capelle, 69621 Villeurbanne, France; 3 Department of Physics, Faculty of Science, King Abdulaziz University, North Jeddah Branch, P.O. Box 80203, Jeddah, 21589 Kingdom of Saudi Arabia; 4 Department of Physics, Faculty of Science, King Abdulaziz University, P.O. Box 80203, Jeddah, 21589 Kingdom of Saudi Arabia; 5 High School of Sciences and Technology of Hammam Sousse, Sousse University, Sousse, Tunisia

**Keywords:** InAs quantum dots, Molecular beam epitaxy, Optical transitions, Photoluminescence, Picosecond time-resolved photoluminescence, Spectroscopic ellipsometry, Localized-state ensemble model

## Abstract

This paper reports on experimental and theoretical investigations of atypical temperature-dependent photoluminescence properties of multi-stacked InAs quantum dots in close proximity to InGaAs strain-relief underlying quantum well. The InAs/InGaAs/GaAs QD heterostructure was grown by solid-source molecular beam epitaxy (SS-MBE) and investigated via photoluminescence (PL), spectroscopic ellipsometry (SE), and picosecond time-resolved photoluminescence. Distinctive double-emission peaks are observed in the PL spectra of the sample. From the excitation power-dependent and temperature-dependent PL measurements, these emission peaks are associated with the ground-state transition from InAs QDs with two different size populations. Luminescence measurements were carried out as function of temperature in the range of 10–300 K by the PL technique. The low temperature PL has shown an abnormal emission which appeared at the low energy side and is attributed to the recombination through the deep levels. The PL peak energy presents an anomalous behavior as a result of the competition process between localized and delocalized carriers. We propose the localized-state ensemble model to explain the usual photoluminescence behaviors. The quantitative study shows that the quantum well continuum states act as a transit channel for the redistribution of thermally activated carriers. We have determined the localization depth and its effect on the application of the investigated heterostructure for photovoltaic cells. The model gives an overview to a possible amelioration of the InAs/InGaAs/GaAs QDs SCs properties based on the theoretical calculations.

## Background

Self-assembled quantum dots (QDs) have been widely investigated for possible applications in optoelectronics due to the nature of three-dimensional carrier confinement and the δ-like density of states. Recently, QD structures were proposed to realize the intermediate band solar cells (IBSCs), which introduce extra photo-carriers through the valence-IB and IB-conduction band absorptions [1]. The GaAs-based IBSCs with QDs that have smaller energy band gap than GaAs form tandem structures which are able to absorb photons at energies lower than the GaAs energy gap resulting in higher energy conversion efficiencies [2]. The formation of QD intermediate band needs a close-packed multiple layer structure of high-density QDs [3, 4]. However, the crystal quality of InAs QDs degrades as the QD layer number increases and layer spacing decreases owing to the buildup of internal compressive strain. The excessive strain will induce dislocations and defects that thread up from the QDs toward the surface. Therefore, the performance of an InAs/GaAs QD SC also degrades as the number of QD layers increases [5]. To overcome these problems, a strain compensation growth technique has been demonstrated with GaAsN, GaAsP, and GaP buffer layer for InAs/GaAs material systems [6–8]. Another technique to overcome these problems is to cover InAs/GaAs QDs layer with a thin InGaAs strain-reduced layer. Compared to InAs/GaAs QDs, this layer causes a redshift to the photo-response due to the presence of a small lattice mismatch between InAs and InGaAs. The temperature-dependent photoluminescence study provides useful information about the multi-stacked InAs/GaAs QDs SC which is of considerable practical and theoretical interest. Classically, the band gap of a semiconductor material reduces monotonically with increasing temperature. Special materials, such as InAs/GaAs QDs, have shown an anomaly in the PL at low temperatures due to thermally activated carrier transfer mechanisms within the ensemble of the quantum dots. However, these abnormalities disappear progressively after post-growth intermixing processes in the InAs/InGaAs/GaAs QD heterostructures as shown by Ilahi et al. [9]. Heterostructures similar to those of the present study have been investigated for their efficiency in photovoltaic applications by Sayari et al. [10]. Many models have been proposed during the last decades, such as the Passler, Vina, and Varshni one. In order to produce reliable devices, temperature behavior of such kind of InAs/InGaAs/GaAs QD heterostructures must be well understood and this is by the use of the best fitting model. We hereby use the Passler classical model corrected to the thermal redistribution coefficient, in order to better understand the observed S-shape temperature dependence of the excitonic band gap. Our study gives rise to a self-consistent precise picture for carrier localization and transfer in an InAs/InGaAs/GaAs QD heterostructure, which is an extremely technologically important energy material for fabricating high-efficiency photovoltaic devices.

## Experimental Details

**Fig. 1:**
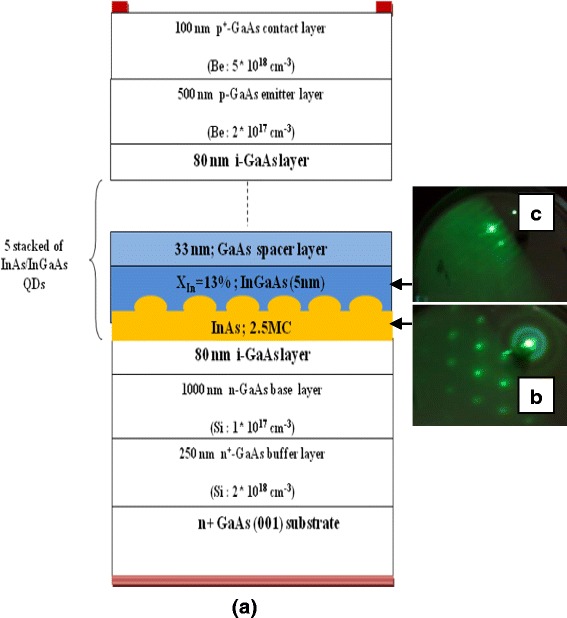
**a** Schematic layer structure of the five-layer stacked InAs/InGaAs QDs SC grown on (001) n^+^-GaAs substrate. **b** RHEED patterned after growth of 2.5 ML of InAs (three-dimensional growth). **c** RHEED patterned during growth of 5 nm InGaAs (two-dimensional growth) [10]

Figure 1 illustrates a schematic diagram of the InAs/InGaAs/GaAs QD heterostructure investigated in our study. The heterostructure consists of five stacks of InAs/In_0.11_Ga_0.89_As/GaAs QD layers sandwiched by 80-nm intrinsic GaAs layer. The epitaxial layers were grown on epiready n^+^-GaAs (100) substrate using solid-source molecular beam epitaxy (SS-MBE) with Riber MBE 32P system. Following oxide desorption, a 250-nm n^+^-doped GaAs buffer with a doping density of 2 × 10^18^ cm^−3^ was grown at 520 °C followed by a 1000-nm n-doped GaAs base layer with a doping density of 10^17^ cm^−3^. The substrate temperature is then lowered and stabilized at 500 °C for the deposition of the intrinsic region. As shown in Fig. 1, the repeated layers consist of 2.5 monolayers (ML) of InAs coverage, 5-nm-thick In_0.11_Ga_0.89_As and 33-nm-thick GaAs. The formation of the QDs was controlled in situ by monitoring the diffraction pattern of high-energy electrons (RHEED). The purpose of the 5-nm-thick In_0.11_Ga_0.89_As is to redshift the emission and absorption spectra, while the 33-nm-thick GaAs acts as a spacer layer. The growth rates for InAs, In_0.11_Ga_0.89_As, and GaAs layers were 0.08 ML/s, 0.78 ML/s, and 0.7 ML/s, respectively, measured by RHEED specular spot oscillations. At the end, a 500-nm p-doped GaAs emitter layer (2 × 10^17^ cm^−3^) followed by a 100-nm GaAs p^+^-doped contact layer (5 × 10^18^ cm^−3^) were grown on top of the heterostructure. Silicon (Si) and beryllium (Be) were used as n- and p-type dopants, respectively. During the growth, the temperature was calibrated by a pyrometer.

Spectroscopic ellipsometry (SE) was performed at room temperature between 1 and 6 eV, using a J.A. Woollam variable angle spectroscopic ellipsometer (VASE) M-2000. The SE measurements were performed at angles of incidence ranging from 45° to 60°. In PL measurements, an argon ion (Ar^+^) laser with a wavelength of 514.5 nm was used as an excitation source to generate electron-hole pairs. The luminescence light from the samples was dispersed by a high-resolution spectrometer and detected by a thermoelectrically cooled Ge photo-detector with a built-in amplifier. For the excitation power-dependent and temperature-dependent PL measurements, the samples were mounted in a closed-cycle, temperature-controlled helium cryostat. The PL spectra were taken in the nominal output power range of 1.5–350 mW and the temperature range of 11–300 K. The time-resolved PL measurements were performed in a variable-temperature (10–240 K), closed-cycle helium cryostat. The 514 nm line was used as an excitation wavelength, from a mode-locked Ti: sapphire picosecond pulse laser at a repetition rate of 80 MHz with a 1.2 ps pulse width.

## Results and Discussions

**Fig. 2:**
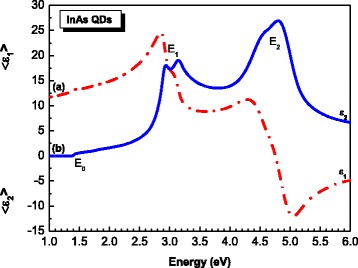
Real (*ϵ*_1_) (*dashed red line*) and imaginary (*ϵ*_2_) (*solid blue line*) parts of dielectric functions of the InAs/InGaAs QD heterostructure obtained from SE measurements [10]

Figure 2 shows the measured real (a) and imaginary (b) parts of the dielectric function of the InAs/InGaAs/GaAs QD heterostructure at 300 K for the energy range 1–6 eV. The real and imaginary parts follow different patterns. The variation of the dielectric function with photon energy indicates that some interactions between photons and electrons in the films are produced in the energy range of 1–6 eV. The two major spectral features are the E_1_ and E_2_ critical point (CP) structures at ~3 and ~4.5 eV, respectively [11, 12]. To quantitatively determine the energy position of the different interband transitions, we took the zero crossing of the second derivative spectrum of the imaginary part of the pseudodielectric function.The different transition energies obtained are summarized in Table 1.

**Table 1:** Comparison between the different CP energies of GaAs and InAs obtained in our previous work and those in Refs. [11, 12]

CP energy	*E*_0_ (eV)	*E*_0_ + Δ_0_ (eV)	*E*_1_ (eV)	*E*_1_ + Δ_1_ (eV)	*E*_0_’ (eV)	*E*_2_ (eV)
Our data: Ref. [10]	1.4	1.75	2.88	3.09	4.39	4.73
GaAs: Ref. [11]	1.42	1.75	2.91	3.14	4.45	4.77
InAs: Ref. [12]	–	–	2.48	2.74	4.39	4.71

**Fig. 3:**
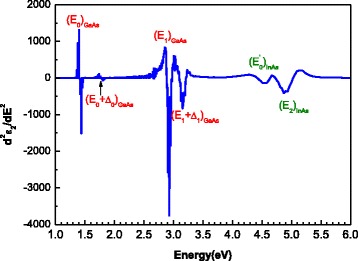
Second derivative spectrum of imaginary part (*ϵ*_2_) of dielectric function as function of the photon energy for the InAs/InGaAs QD heterostructure. The transition energies arising from InAs QD layers and GaAs layers are indicated [10]

Figure 3 shows the second energy derivative spectrum of the imaginary part of the pseudodielectric function shown in Fig. 2. The two peaks at 2.9 and 3.1 eV correspond, respectively, to the *E*_1_ and *E*_1_ + Δ_1_, interband transitions in GaAs. However, the two closely positioned peaks at about 4.4 and 4.7 eV are caused by the CP transitions *E*_0_’ and *E*_2_, respectively, in InAs QD layers [12]. We note that the contribution of the *E*_1_ + Δ_1_ CP energy (2.74 eV) [12] of InAs to the *E*_1_ one (2.91 eV) [11] of GaAs cannot be excluded due to the small difference between the two energy values. At low energy, the band gap of GaAs is clearly distinguishable in the *ε* spectrum at about 1.4 eV. Also, the second energy derivative spectrum (Fig. 3) shows an interband transition at 1.75 eV which corresponds to the *E*_0_ + Δ_0_ CP energy of GaAs [11]. It is known that *ε*_2_ is a gauge and measure of material quality; the highest value implies the most abrupt interface [13]. According to literature, *ε*_2_ values of about 25, the highest value being 26.8 in our case, obtained in the region of the *E*_2_ band gap near 4.7 eV, indicate the high quality of materials forming the InAs/InGaAs/GaAs QD heterostructure grown by SS-MBE.

**Fig. 4:**
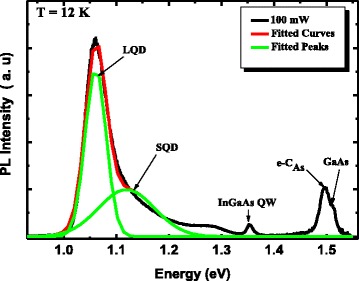
PL spectrum measured at low temperature (12 K) from the five-layer stacked InAs/InGaAs QD heterostructure. A line shape analysis of spectra proves that the QD PL signal is a convolution of two Gaussian-shaped peaks as shown by *solid lines*

**Fig. 5:**
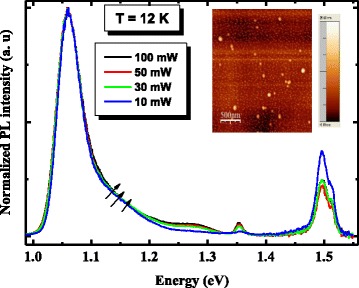
Excitation power-dependent normalized PL spectra from the InAs/InGaAs QD heterostructure measured at 12 K

Figure 4 shows the PL spectrum of the active region in the InAs/InGaAs/GaAs QD heterostructure with an excitation power of 100 mW at low temperature (12 K). Obviously, the spectrum presents an asymmetric shape located at the high-energy side and which can be deconvoluted in two sub-bands by Gaussian fitting [14]. Considering the dependence of the quantum confinement potentials on the dot size, the most intense peak located at 1.06 eV is attributed to the emission from the ground states of larger QDs (LQDs), while the higher energy peak at 1.11 eV refers to the emission from the ground states of smaller QDs (SQDs) [15]. Thus, at very low excitation power and low temperature, we deduce that the asymmetric shape is due to luminescence originating from a bimodal size distribution of dots [16]. In addition, peaks originating from InGaAs quantum well layer, the recombination between electrons in the GaAs conduction band and holes at the carbon acceptor level (e-C_As_) [17] and GaAs band gap are seen around 1.35, 1.49, and 1.51 eV, respectively. To confirm this attribution to the asymmetric shape, we carried out PL measurements at different laser power ranging from 10 to 100 mW. We also performed AFM measurements on an uncapped structure similar to the investigated one. From Fig. 5, it is clear that the heterostructure has a power-independent PL shape. Apart from the highest excitation spectrum, the PL intensity and line width of the heterostructure high-energy PL peak are not significantly changed. Also, the energy separation between the two PL peaks (Fig. 5) is around 50 meV. As expected, the AFM image demonstrates that the QDs in the fifth layer possess a bimodal size distribution with a whole QDs density of 7 × 10^10^ cm^(−2). Assuming that the low-energy side peaks of the heterostructure correspond to the ground state of large QDs (LQDs), we can say that the high-energy peaks at high power appears to be the result of the ground states of relatively small QDs (SQD).

**Fig. 6:**
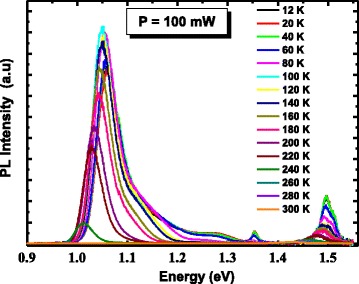
Temperature-dependent PL spectra from the InAs/InGaAs QD heterostructure measured at an excitation power of 100 mW

**Fig. 7:**
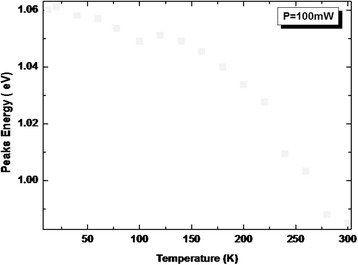
LQD PL peak energies of the investigated InAs/InGaAs QD heterostructure measured at various temperatures

To get an insight in the PL recombination mechanisms, temperature-dependent PL measurements are performed on the heterostructure from 10 to 300 K and depicted in Fig. 6. Figure 7 shows the PL energy position of the principal peak associated with emission from LQDs. This peak shows an abnormal temperature dependence between 10 and 100 K compared to other III–V ternary alloys such as conventional GaAlAs [18]. In this temperature range (region (i)), a redshift of around 12 meV is observed. This shift is due to recombination of excitons via the localized sates within the ensemble of the inhomogeneously distributed LQDs. As the temperature increases within this range of temperatures, carriers are thermally activated and transferred from the smaller to the larger QDs within the ensemble, where they eventually recombine radiatively. This makes the observed phenomena likely to originate from the large dot size dispersion in our structure (see AFM inset). The characteristic temperature in which the two recombination processes (localized and delocalized carriers) participate equally in the PL signal is denoted as *T*_loc/deloc_. Then, between 100 and 120 K (region (ii)), the peak energy increases. This is due to the escape of carriers from the shallower states to the higher ones. The characteristic temperature is denoted as *T*_escape_. At even high temperature (region (iii)), the excitons are totally delocalized and a band-to-band recombination is recovered.

For further understanding the recombination process in InAs/InGaAs/GaAs multi-stacked QDs, we have studied the time-resolved PL using the photocounting time-correlated technique. It was predicted theoretically that the exciton decay lifetime of QDs is sensitive to temperature [19]. Experimental measurements have shown that the lifetimes are indeed a constant of temperature below a critical temperature [20]. Markus et al. [21] reported a constant lifetime of about 950 ps over a wide range of temperature within the experimental error.

**Fig. 8:**
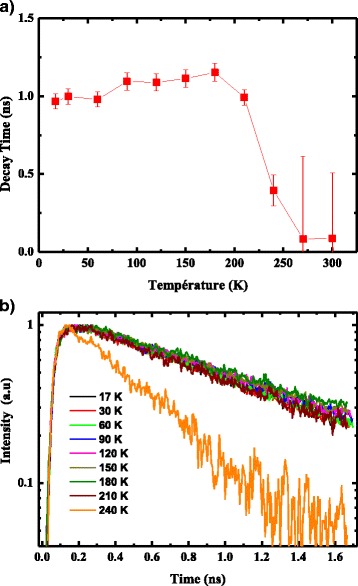
**a** Decay time of the LQDs peak versus the sample temperature for a wavelength excitation of 514 nm. **b** Typical photoluminescence decay intensity versus the temperature of the LQD peak measured at an excitation wavelength of 514 nm

Figure 8a presents the PL decay spectrum, between 17 and 240 K for a detection energy fixed at 1.06 eV and an excitation energy (*λ*_exc_ = 514 nm). Those spectra are well fitted theoretically by a mono-exponential function, with a decay time of ~1000 ps in lower temperature. This slow decay time, compared to III–V semiconductor thin films [22], is a signature of the presence of localized states [23], and the carriers’ recombination at LQD peak should be a purely radiative one. Indeed, at low temperature, photo-generated electrons and holes, before they recombine, take time to form excitons and relax their energy to be captured by the shallow localized states. These phenomena lead to slow decay time. The temperature effect on the PL decay time has been studied and shows the presence of two different regimes as represented in Fig. 8b [24]. We remark that the decay time associated to the lower energy of the PL band (LQDs (1.06 eV)) is almost constant (1000 ps) up to 140 K, and then, it decreases as the temperature increases.

## Theoretical Approach

In order to understand the anomalous temperature dependence of PL, the PL peak position has been investigated using the LSE model developed by Li et al. [25, 26]. Indeed, this quantitative model provides a satisfactory explanation for the anomalous spectral features of the localized-state luminescence previously observed in several III–V materials such as boron-based B(In)GaAs/GaAs [27, 28] alloys and InGaAs/GaAs MQWs [29, 30]. The model assumed that the localized state has a Gaussian-type energy distribution for density of states given by:

1 $$ \rho (E)={\rho}_o{e}^{-{\left(\frac{E-{E}_{\mathrm{ch}}}{2{\sigma}^2}\right)}^2} $$

Where *σ* and *E*_*ch*_ are the broadening parameter for the distribution of the localized states (localization depth) and the barrier level that the carriers must overcome to transfer, respectively. Under quasi steady state, the dynamics of the localized excitons can be described by the following rate equations:

2 $$ \frac{dN\left( E, T\right)}{dt}= G(E)+\frac{\gamma_c N\hbox{'}\left( E, T\right)\rho (E)}{\Lambda}-\frac{N\left( E, T\right)}{\tau_{\mathrm{tr}}}{e}^{\frac{E-{E}_a}{K_B T}}-\frac{N\left( E, T\right)}{\tau_{\mathrm{r}}}=0 $$

The parameters *τ*_r_ , *τ*_tr_, *γ*_c_ , *K*_B_, Λ, and *N* ' represent, respectively, the carrier recombination time (radiative lifetime), the carrier transfer time (non-radiative lifetime), recapture coefficient, Boltzmann constant, total number of localized states, and the total number of carriers that are thermally activated away from the localized states. *G* (*E*) represents the rate of carrier generation. The quantity $\frac{\gamma_c N\hbox{'}\left( E, T\right)\rho (E)}{\Lambda}$ is the number of carriers re-captured by the localized states per unit time. The third term on the right gives the thermal escape rate of the localized carriers. The last one represents the de-population rate of carriers due to the radiative recombination. The carrier population density of localized carriers is proportional to the distribution function and density of states of localized carriers. In fact, the solution of Eq. (2) can be described by Eq. (3).

3 $$ N\left( E, T\right)=\frac{\rho_o{e}^{-{\left(\frac{E-{E}_0}{2{\sigma}^2}\right)}^2}}{\left[\frac{\tau_{\mathrm{tr}}}{\tau_{\mathrm{r}}} + exp\left(\frac{\left( E-{E}_{\mathrm{ch}}\right)}{K_{\mathrm{B}} T}\right)\right]} $$

where *E*_0_ is the central energy. Mathematically, the temperature dependence of the peak position due to carrier thermal redistribution within the localized states determined from $\frac{\partial N\left( E, T\right)}{\partial t}=0$ is given by:

4 $$ E(T)={E}_0- x(T){K}_B T $$

Where *x* (*T*) is the numerical solution of the nonlinear Eq. (5):

5 $$ x{e}^x=\left[{\left(\frac{\sigma}{K_b T}\right)}^2- x\right]\left(\frac{\tau_{\mathrm{r}}}{\tau_{\mathrm{tr}}}\right) exp\left[\frac{\left({E}_0-{E}_{\mathrm{ch}}\right)}{K_{\mathrm{B}} T}\right] $$

Equation 5 has only one solution for $0< x<{\left(\frac{\sigma}{K_b T}\right)}^2$. In high-temperature region, the approximated solution is ${\left(\frac{\sigma}{K_{\mathrm{B}} T}\right)}^2$. Equation (5) reveals the band-tail model proposed by Eliseev et al. [31]:

6 $$ E(T)\approx {E}_0-\frac{\sigma^2}{K_B T} $$

**Fig. 9:**
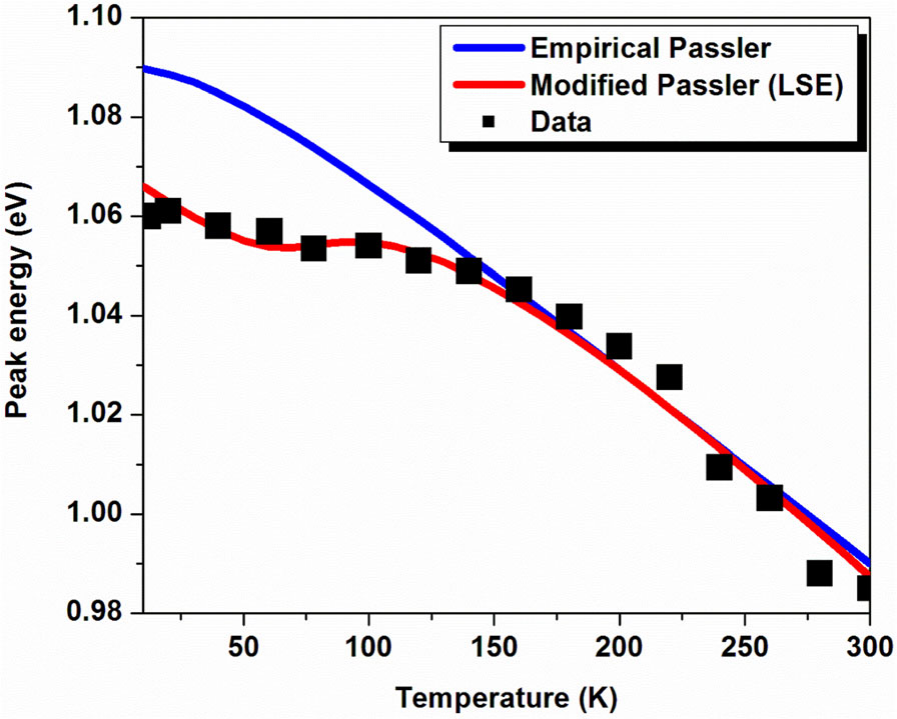
Temperature-dependent photoluminescence evolution of the LQD peak (*solid black squares*) fitted using the empirical Passler law (*blue solid line*) and the modified Passler relation truth to the LSE model (*red solid line*)

It is known that the band gap of an idealized semiconductor material is usually described by the Passler empirical formula [32]. Taken into account the correction due to the thermal redistribution coefficient, the variation of the peak position of luminescence using LSE model described by Eq. (7):

7 $$ E(T)={E}_0-\frac{\alpha \theta}{2}\left[\sqrt[ P]{1+\left(\frac{2 T}{\theta}\right)}\kern0.5em -1\right]- x(T){K}_B T $$

where *θ* is a characteristic temperature parameter which was expected to be comparable with the Debye temperature *θ*_D_. For *T*>>*θ*, we see that α represents just the limit of the magnitude of the first derivative, ${\frac{dEg(T)}{dT}}_{T\to \infty }$. The exponent “p” is related to the shape of the underlying electron-phonon spectral function [33]. The model provides a good fit to the experimental evolution which is confirmed by Fig. 9. The fitting parameters are summarized in Table 2.

**Table 2:** Parameters used to fit the energy evolution using empirical Passler (a) and modified Passler (b) model (LSE)

	*E*_0_ (eV)	*σ* (eV)	*E*_ch_-*E*_0_ (eV)	α (10^−4^ eV/K)	*p*	*τ*_r_/*τ*_tr_	*θ* (K)
LSE model	1.066	19 × 10^−3^	4 × 10^−3^	5	2.5	10	110
Empirical Passler	1.090	–	–	4.1	2.5	–	110

**Fig. 10:**
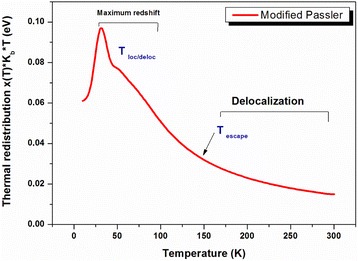
Temperature-dependent thermal redistribution determined numerically. The characteristic temperatures (*T*_loc/deloc_ and *T*_escape_) are indicated with respect to the localization-delocalization process

**Fig. 11:**
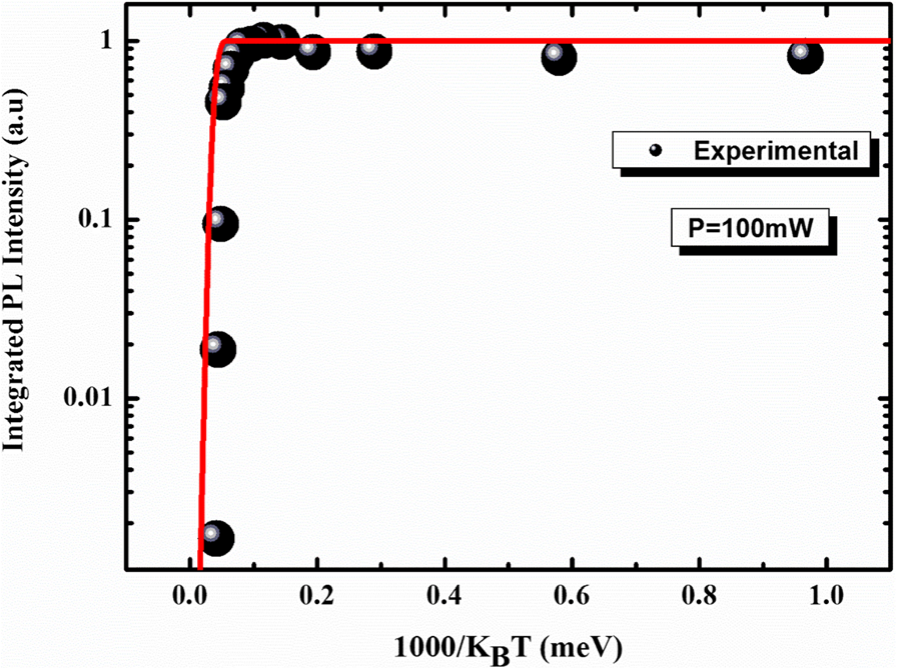
Arrhenius fitting of the investigated sample. The normalized integrated intensity (*black circles*) is fitted with three activation energies (*red solid line*)

**Fig. 12:**
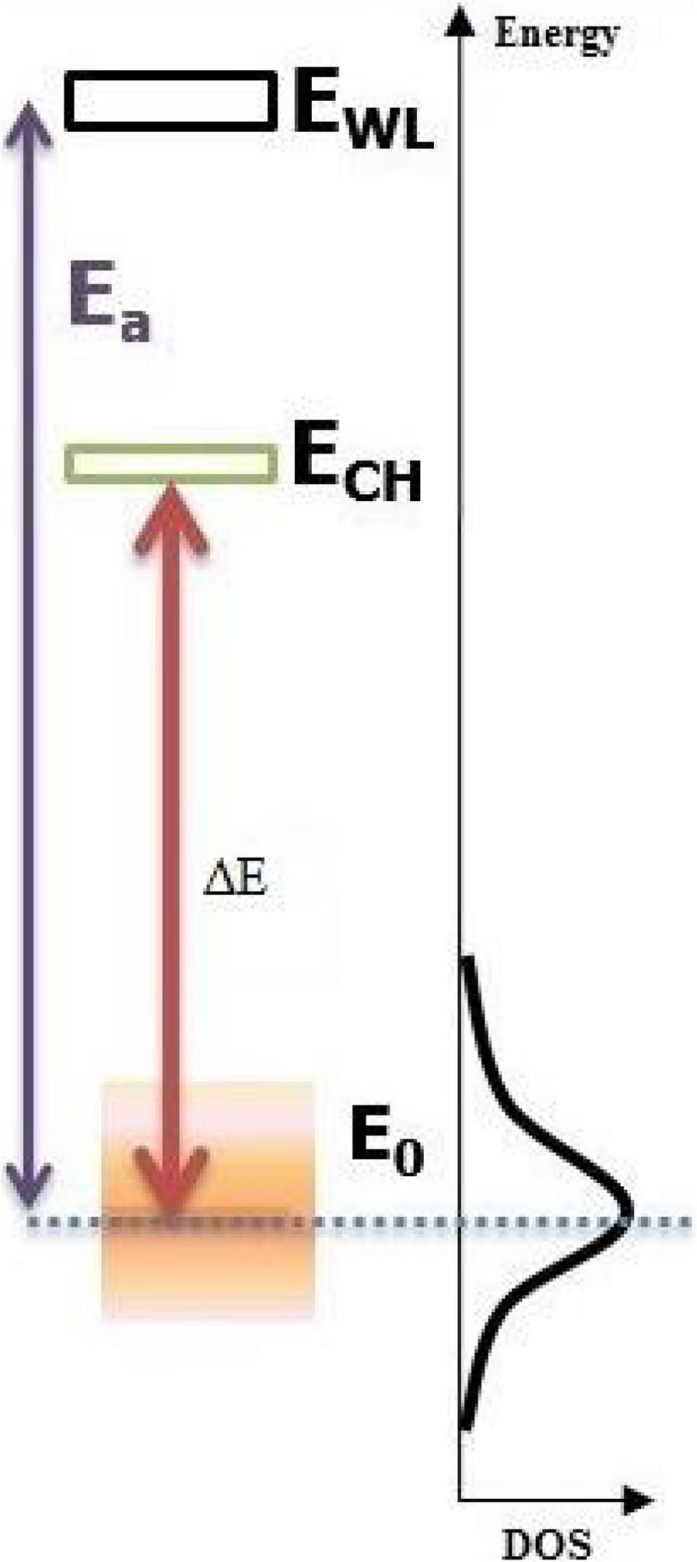
Schematic of the QD distribution of localized electron/hole (exciton) states (*WL* wetting layer, *CH* carrier transfer channel)

The PL peak energy strongly depends on the thermal redistribution represented in Fig. 10. This last indicates a rapid increase in the range of cryogenic temperature. The maximum of thermal redistribution corresponds to the maximum of redshift in the energy evolution (~50–100 K). In the region of high PL temperature, the thermal redistribution decreases exponentially and tends to cancellation as from 150 K it starts the delocalization process and the return to band-to-band transitions. Also, we can observe this when the classical and the modified curves are superposed (Fig. 9).The exponent “p” indicates that the contribution of the longitudinal acoustic (LA) phonons is more significant than the contribution of the longitudinal optical (LO) phonons. This contribution appears to be dominant in the region of high PL temperature where the emission is assisted via phonons. The bimodal distribution process opens a coupling channel between QDs which is represented by delocalized electron and hole states separated by an energy *E*_ch_. The origin of this coupling channel is still a subject of controversy [34–36]. However, the coupling channel can be viewed as the intermediate states existing between two-dimensional WL and zero-dimensional QD states [37]. So, it can be imagined that the carriers in the QD states can be more easily thermally excited to the coupling channel than the WL due to the smaller activation energy needed, then transferred to their neighboring QDs within a finite distance. It appears like the Fermi-Dirac level in the Fermi-Dirac distribution. This energy *E*_ch_ is smaller than the activation energy *E*_a_ extracted from the Arrhenius diagram (Fig. 11). The reason why *E*_a_ is larger can be explained by the fact that carriers need larger energy to reach the wetting layer (WL) as shown schematically in Fig. 12. Moreover, the magnitude of the difference Δ*E* = *E*_ch_ − *E*_0_ plays a more significant role in determining abnormal temperature dependence of luminescence of localized carriers. We should note that the two cases, *E*_ch_ − *E*_0_ > 0 and *E*_ch_ − *E*_0_ < 0, exist from a physical point of view, but it is usually assigned as “positive” thermal activation energy. In our case, this implies that *E*_ch_ is 4 meV below *E*_0_ in which localized carriers are thermally activated to states (or sites in real space) with higher energies. It decreases compared to a single InAs QD layer with In_0.15_Ga_0.85_As strain reducing underlying layer [10]. The potential fluctuation depth assigned by *σ* is a result of size distribution inhomogeneity of QDs. The potential depth is found to be 19 meV. It decreases by increasing the number of stacks of InAs/In_0.11_Ga_0.89_As/GaAs QDs. As a result, we can deduce that the decrease of potential depth increases the structure efficiency compared to the one layer of InAs/InGaAs/GaAs QDs studied by Ilahi et al. and Helmi et al. [10, 36].

## Conclusion

In conclusion, we have successfully fabricated GaAs-based SC with a multi-stack of InAs QDs by capping an InGaAs layer on the QDs and inserting GaAs spacer layers. The two major spectral features observed in the dielectric function spectra of the InAs/InGaAs/GaAs QD heterostructure at 3 and 4.5 eV are attributed to the *E*_1_ and *E*_2_ CP structures of GaAs and InAs, respectively. The PL spectrum of the InAs QDs in the GaAs matrix is intense and presents an asymmetric shape, which indicates the growth of a high-quality, multi-stacked InAs QD structure. The contribution of larger and relatively smaller QDs to the PL spectrum is also demonstrated. The luminescence measurements were successfully modeled and re-interpreted using the developed LSE model. The theoretical study has quantitatively interpreted the observed temperature-dependent spectra, and has shed light on the complicated spontaneous emission mechanisms in multi-stacked InAs/InGaAs/GaAs QDs, based on the fitting parameters. This study suggests a way to improve the efficiency of InAs/GaAs QD structures for their use in photovoltaic applications. These results help to improve the understanding of the temperature-dependent carrier dynamics in strain-engineering QDs in order to improve the efficiency of the investigated structure. Further to this work, we will study the effect of orientation as well as the increase in the number of InAs/GaAs QDs of the multi-stack structure on the localization depth.
